# Catalytic anti-oxidative stress for osteoarthritis treatment by few-layered phosphorene

**DOI:** 10.1016/j.mtbio.2022.100462

**Published:** 2022-10-15

**Authors:** Xingyu Zhang, Yanling You, Yaying Sun, Xiang Guo, Han Lin, Ming Zong, Jianlin Shi

**Affiliations:** a Department of Sports Medicine, Shanghai General Hospital, Shanghai Jiao Tong University School of Medicine, Shanghai, 200080, PR China; b Department of Clinical Laboratory, Shanghai East Hospital, Tongji University School of Medicine, Shanghai, 200120, PR China; c State Key Laboratory of High Performance Ceramics and Superfine Microstructure, Shanghai Institute of Ceramics Chinese Academy of Sciences, Shanghai, 200050, PR China; d Department of Sports Medicine, Huashan Hospital, Fudan University, Shanghai, 200040, PR China; e Department of Orthopedics, Shanghai Changzheng Hospital, Second Affiliated Hospital of Naval Medical University, Shanghai, 200003, PR China

**Keywords:** Phosphorene, In-situ catalysis, Anti-inflammatory, Osteoarthritis, Nanocatalytic medicine

## Abstract

As one of the most common representations of articular cartilage damage, osteoarthritis (OA) is characterized by the apoptosis and dysfunction of chondrocytes as well as the progressive degradation of extracellular matrix, of which the main components are glycosaminoglycan and type Ⅱ collagen. Few-layered phosphorene (FLP) has been attracting great attentions in biomedical fields owing to the excellent capability of in-situ catalysis for scavenging oxidate-associated molecules, especially the reactive oxygen species (ROS) and reactive nitrogen species (RNS). Herein, FLP has been fabricated and employed for articular cartilage protection by means of deleting oxidate-associated molecules. The *in vitro* results show that as low as 200 ​μg/mL FLP is capable of diminishing oxidative damages on the osteoarthritic chondrocytes through the efficient elimination of ROS, H_2_O_2_ and NO. Meanwhile, the cartilage matrix protection has also been achieved at 200 ​μg/mL FLP by the uniform restoration of glycosaminoglycan and type Ⅱ collagen. FLP enables the nanocatalytic treatment for the overloaded oxidative stress in the injured articular cartilage and represents a promising alternative for osteoarthritis therapy.

## Graphical abstract

**Figure:**
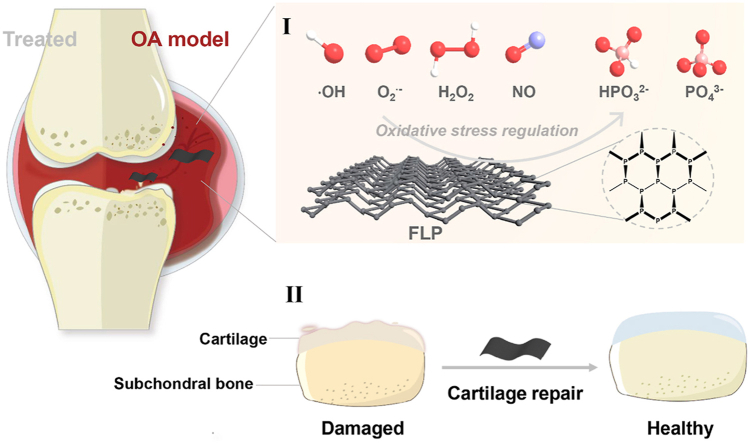

## 1 Introduction

Articular cartilage lacks blood vessels, nerves and lymph supplies, which would lead to intrinsic limitations of self-repair and regeneration in the aging process and joint injury, including various common pathological conditions like osteoarthritis (OA), rheumatoid arthritis and local cartilage defect [1]. Although multiple treatments such as conventional drug administrations, microfracture technique, cartilage transplantation surgery and stem cell-based therapies have been performed in surgical clinical trials, the optimal solution in repair and regeneration of articular cartilage is still vague [2,3]. Injured cartilage would not only induce DNA damage and protein denaturation, but accelerate the degradation of the extracellular matrix due to the considerable generation of reactive oxygen species (ROS) that responds to the inflammatory-related signals and chemicals, among which hydrogen peroxide (H_2_O_2_) features the most common and abundant category [[4], [5], [6]]. In addition, the over-production of nitric oxide (NO) surrounding the cartilage and synovial tissues during the progressive stage of arthritis conversely promotes the destruction of cartilage and synovium in the injured joint [[7], [8], [9]]. Therefore, it is of great importance to efficiently restrict the production of these oxidate-associated molecules and repair the damaged articular cartilage.

The rapid development of clinical biomedicine and nanobiotechnology has furnished the emergence of diverse inorganic nanosystems, which offers multiple therapeutic routines as potential alternatives in combating various pathological abnormalities, especially in orthopedic diseases [10,11]. Currently, great efforts for cross-disciplinary research frontier have been focused on biomedical applications of two-dimensional (2D) nanomaterials, a newly emerging subtype of nanomaterials with ultrathin layer-structured topology [[12], [13], [14], [15]]. As a new family of 2D nanomaterials, few-layered phosphorene (or black phosphorus) has been arousing much interest in tremendous biomedicine fields such as drug nanocarriers [16,17], tumor theranostics [[18], [19], [20]], biosensors [20], and bone formation [16,[20], [21], [22]]. For instance, phosphorene nanosheet was previously applied in specific drug delivery for depression therapy, photothermal and photodynamic treatments for tumor immunotherapy as well as biomineralization and repair for bone defect [[23], [24], [25], [26]]. Nevertheless, the therapeutic practice of phosphorus-based nanomaterials for articular cartilage restoration and injury repair is rarely demonstrated in the previous studies [27]. Taken together, 2D structural phosphorene featuring high surface-to-volume ratio, flexibility of modification, and ample physicochemical properties [22], and it marks a promising alternative for cartilage protection by eliminating the oxidate-associated molecules.

**Scheme 1:**
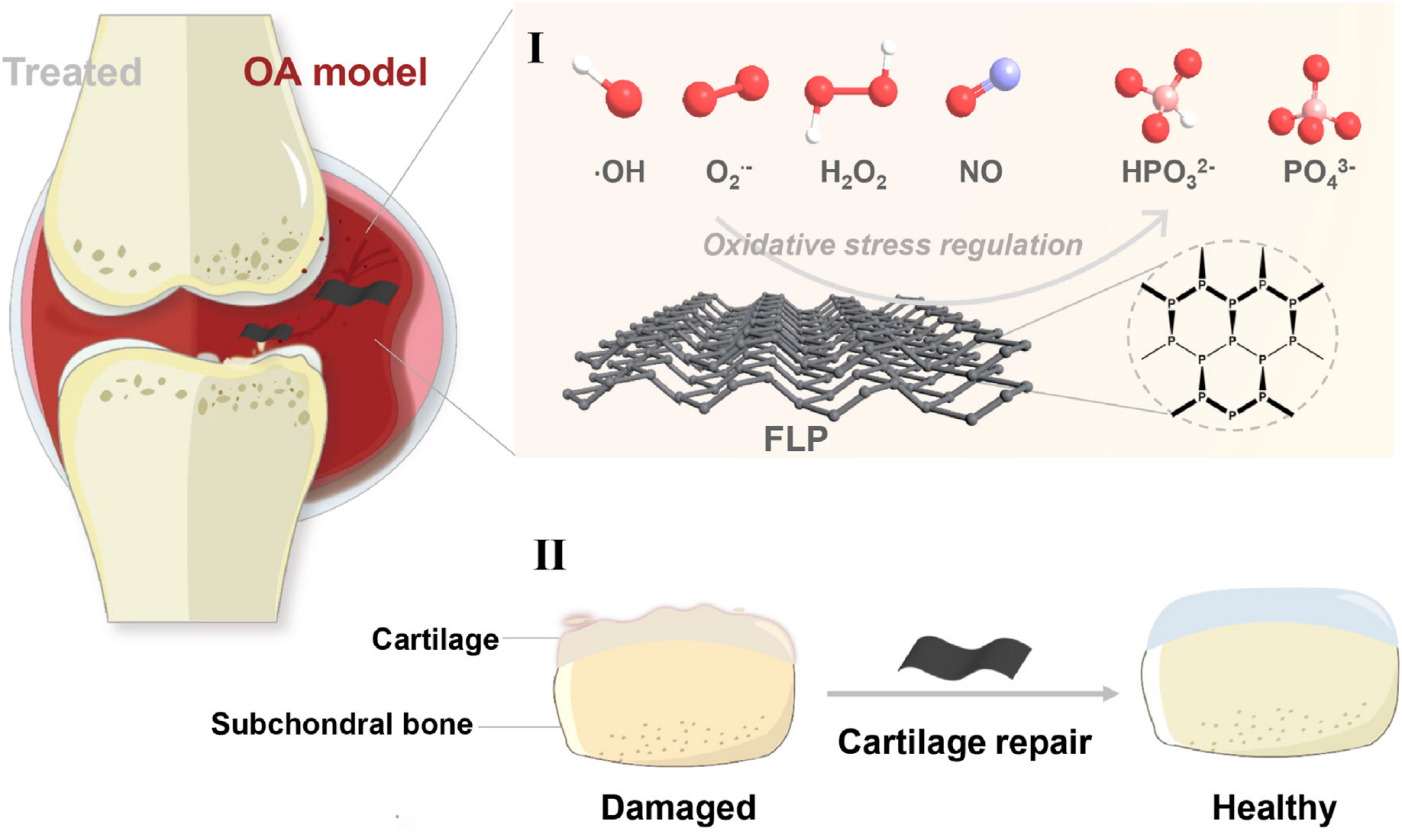
Schematic illustration of the therapeutic mechanisms of FLP in OA treatment.

Herein, few-layered phosphorene (FLP) was applied for the injured cartilage tissue with a rat model of osteoarthritis, and further investigated of its efficacy for cartilage protection. The injured chondrocytes were firstly prepared through the stimulation of inflammatory factors, and co-incubated with FLP to determine the appropriate working concentrations. After the dosages of 50 ​μg/mL and 200 ​μg/mL FLP were determined, the survival analysis and measurements of oxidate-associated molecules of the injured chondrocytes were conducted by co-incubation with FLP for 48 ​h. Afterwards, by using *in vivo* rat model of osteoarthritic injured cartilage, the cartilage protection ability of FLP was validated through the staining of extracellular matrix consisting of glycosaminoglycan and type II collagen (Scheme 1). Therefore, this study provides convincing evidences of FLP-based cartilage protection in the joint diseases.

## 2 Results and discussion

### 2.1 Synthesis and characterization of FLP

**Fig. 1:**
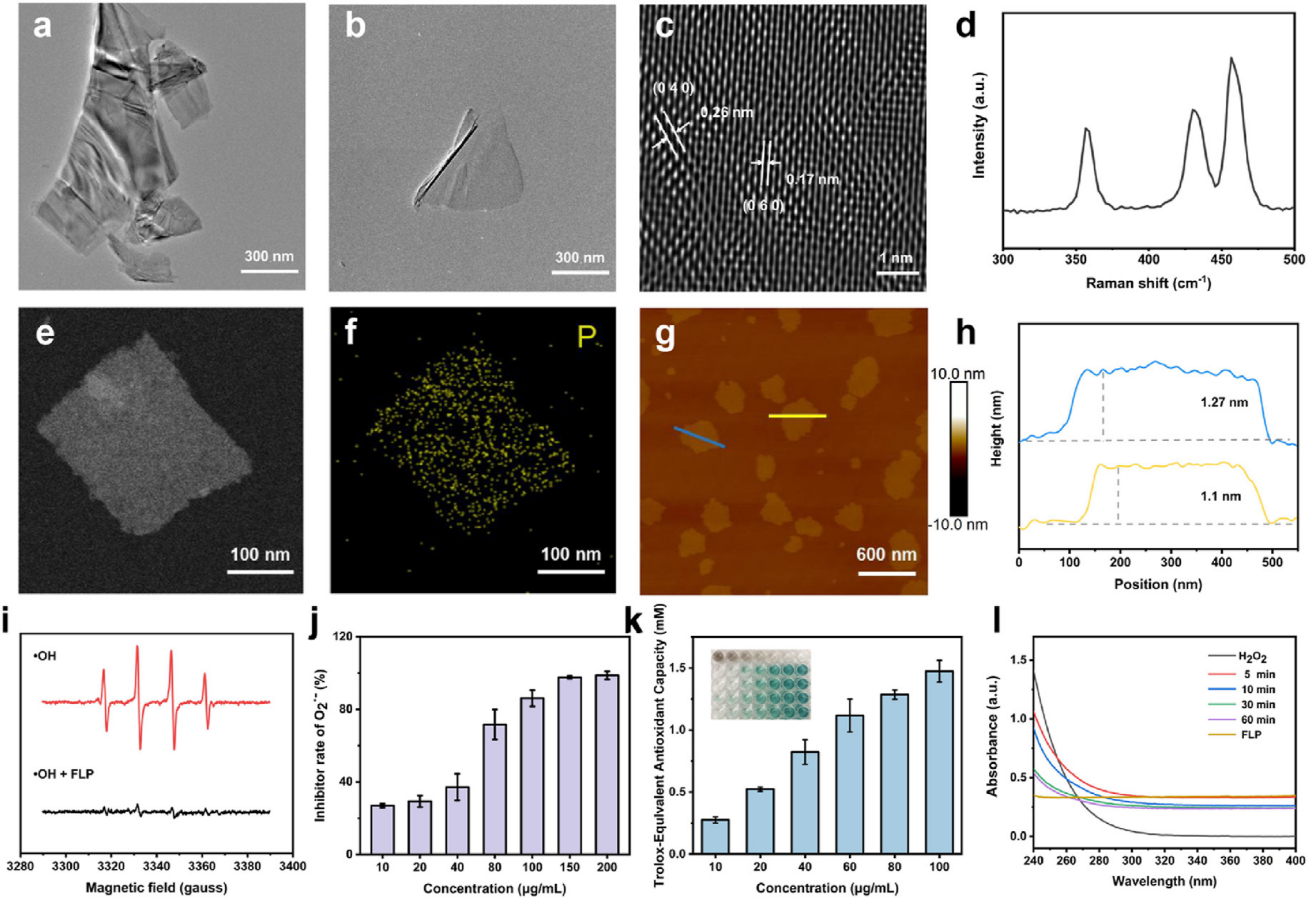
Characterization and ROS scavenging ability evaluation of FLP. (a and b) TEM images of FLP after ultrasound treatment for varied time (a:12 ​h, b:18 ​h). (c) HAADF-STEM image of FLP. (d) Raman spectra of FLP. (e and f) STEM image of FLP and the corresponding EDS elemental mapping. (g) AFM image of FLP and (h) thickness analysis of FLP. (i) ESR spectra of FLP reacted with ^•^OH. (j) Superoxide anion scavenging assay of FLP at varied concentrations. (k) Antioxidative capacity evaluation of FLP. (l) The absorbance changes of H_2_O_2_ reacted with FLP at varied time points.

In this study, the few-layered phosphorene (FLP) was synthesized by an ultrasonication-assisted mechanical exfoliation method using bulk black phosphorus as a precursor. TEM images display freestanding few-layered or monolayer nanosheets featuring the planar morphology with lateral size of hundreds of nanometers after ultrasonication treatment for 18 ​h (Fig. 1a and b). The crystalline FLP was further investigated by scanning transmission electron microscopy with high-angle annular dark field (HAADF-STEM). It could be found that a clear crystal lattice spacing of 0.17 and 0.26 ​nm corresponded to lattice planes of (0 6 0) and (0 4 0) of as-prepared FLP (Fig. 1c). The elemental mapping of FLP suggests a typical monoelemental layered microstructure (Fig. 1e and f). AFM results match the few-layered or monolayered structure of FLP with a thickness of 1.0–1.3 ​nm (Fig. 1g and h). Raman scattering spectrum shows three characteristic Raman peaks at ∼356.7 ​cm^−1^, ∼430.1 ​cm^−1^, and ∼456.7 ​cm^−1^, in accord with the three vibrational modes of A^1^g, B^2^g, and A^2^g, respectively (Fig. 1d). The above characterization results demonstrated the successful preparation of FLP.

### 2.2 Antioxidative efficiency evaluation

As one of the cytotoxic ROS, hydroxyl radicals (^•^OH) were generated by Fenton reaction and captured by DMPO, which generated a typical 1:2:2:1 signal peak in ESR spectroscopy. Obviously, the significantly attenuated signal of ^•^OH peaks could be seen upon the addition of FLP, demonstrating the high ^•^OH scavenging capability (Fig. 1i). Additionally, the O_2_^•−^ scavenging capability was assessed by color reaction of WST-1 with O_2_^•−^, for the reaction product featuring the characteristic absorption at 450 ​nm. It is found that the inhibition rate of O_2_^•−^ significantly increased with elevated FLP concentration and reached almost 100% when the concentration up to 150 ​μg/mL, implying that the O_2_^•−^ can be effectively eliminated by FLP (Fig. 1j). Considering that the excessive oxidative stress is closely related to cartilage injury [28], the potential antioxidative effectiveness of FLP would be assessed with the total antioxidant capacity assay kit. The generation of ^•^ABTS+, the radicals oxidized from 2′-azinobis (3-ethylbenzthiazoline-6-sulfonic acid), was suppressed by the increment of FLP while producing the characteristic ABTS decolorization assay. As depicted in Fig. 1k, dose-dependent antioxidative activity was demonstrated, where the efficacy of 100 ​μg/mL FLP was equivalent to 1.4 ​mM Trolox. Notably, the antioxidant capacity ratio of Trolox/glutathione/ascorbic acid was 1/1.09/1 through the ABTS assay. It has been known that H_2_O_2_ features another primary source of cellular ROS [29]. In general, the elimination capacity of H_2_O_2_ was assessed via detecting its characteristic absorption at 240 ​nm. Fig. 1l shows that the absorbance of mixture at 240 ​nm gradually declined over time, indicating a time-dependent increase of H_2_O_2_-inhibiting effect. It is expected that such FLP possessed the superb ROS scavenging ability, which could be employed as an emerging antioxidative nanoagent.

### 2.3 Chondrocyte activation and FLP concentration screening

To investigate the therapeutic efficacy of FLP on cartilage restoration and osteoarthritis treatment, the osteoarthritic injured chondrocytes were prepared with the stimulation of IL-1β according to the previous studies [[30], [31], [32], [33]]. Fig. S1a shows that the expression of CD54 (red fluorescence) in the normal chondrocytes was lower than that in the IL-1β-induced injured chondrocytes (Fig. S1b). Meanwhile, the augmented ratio of CD54/DAPI (red/blue) fluorescence intensity of the injured chondrocytes also revealed a rising expression of CD54 with the stimulation of IL-1β when compared with the normal cells (Fig. S1c). The previous study showed that CD54 expression would be activated and elevated in cartilage injury and inflammatory arthritis [34], thus the over-expression of CD54 in this study suggested the activation of the chondrocytes and the early-stage damage of the cartilage extracellular matrix. The above results implied the successful establishment of the *in vitro* model of the injured chondrocytes.

Afterwards, the appropriate working concentrations of FLP for further practices were determined by a gradient analysis. An elevated concentration series of FLP from 0.01 to 200 ​μg/mL was co-cultured with the injured chondrocytes and examined with the standard Cell Counting Kit-8 (CCK-8) assay. There were no significant inhibition effects on the cell viabilities when the concentrations were less than 0.40 ​mg/mL, and 50 as well as 200 ​μg/mL FLP exhibited the highest viabilities among these examined concentrations (Fig. S1d). Therefore, two FLP concentrations (50 ​μg/mL and 200 ​μg/mL) were selected and applied for following therapeutic experiments.

### 2.4 Survival analysis of FLP-treated injured chondrocytes

**Fig. 2:**
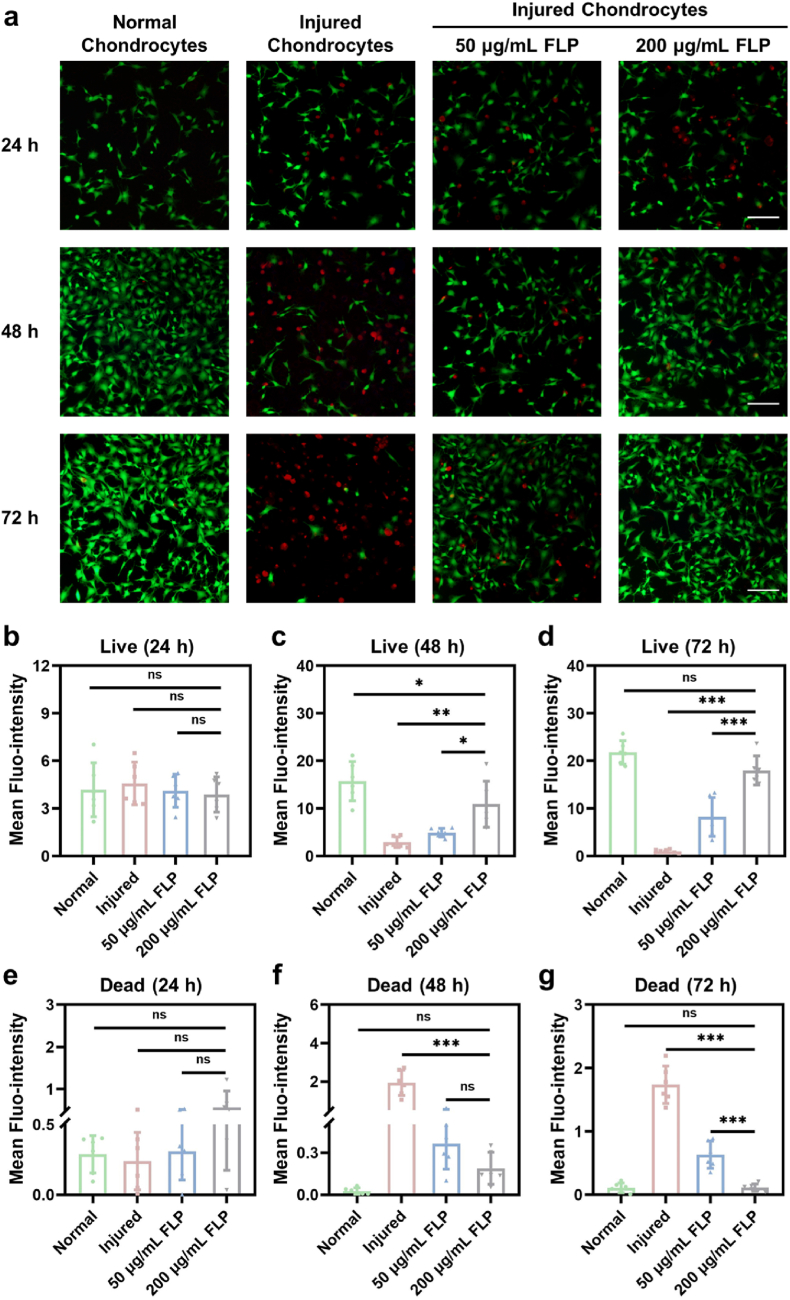
The survival analysis of the injured chondrocytes after FLP treatment. a) Live and dead chondrocytes stained by Calcein/PI assay kit after 24 ​h, 48 ​h and 72 ​h incubation. Green fluorescence represented the live cells while red fluorescence represented the dead cells (Scale bars, 200 ​μm). b-d) Semiquantitative measurement for live cells at 24 ​h, 48 ​h and 72 ​h (n ​= ​6). e-g) Semiquantitative measurement for dead cells at 24 ​h, 48 ​h and 72 ​h (n ​= ​6). ∗P ​< ​0.05, ∗∗P ​< ​0.01, ∗∗∗P ​< ​0.001 and ns meant no significance. All data were presented as mean ​± ​SD. (For interpretation of the references to color in this figure legend, the reader is referred to the Web version of this article.)

Survived chondrocytes in osteoarthritis are necessary to maintain the degenerated cartilage extracellular matrix, and they will encounter injury, degradation and apoptosis [35]. Therefore, in this work, the survival of injured chondrocytes after incubation with FLP was stained with Calcein-AM and PI for cell live/dead observation. As shown in Fig. 2a, with the treatment of 200 ​μg/mL FLP, the green fluorescence of the survived injured chondrocytes gradually increased over time, while the fluorescence of the injured cells without FLP treatment significantly decreased. Furthermore, the green fluorescence of 200 ​μg/mL FLP-treated chondrocytes was also higher than 50 ​μg/mL FLP-treated cells. Moreover, based on the quantitative analysis, the mean fluro-intensity of the chondrocytes was 8.23 ​± ​4.09 as FLP of 50 ​μg/mL versus 18.01 ​± ​3.03 as FLP of 200 ​μg/mL in 72 ​h ​(P ​< ​0.001) (Fig. 2d), when Fig. 2b showed no differences of fluorescence among these groups in 24 ​h and Fig. 2c showed that 200 ​μg/mL FLP-treated fluro-intensity has not resumed to that of the normal cells in 48 ​h. Hence, 200 ​μg/mL of FLP could promote the proliferation of the survived injured chondrocytes, and 50 ​μg/mL FLP just partially maintained the injured cells.

In addition, the dead chondrocytes after IL-1β stimulation were reported by the presence of red fluorescence. After incubation with 200 ​μg/mL of FLP for 72 ​h, the red fluorescence of the injured chondrocytes evidently reduced in comparison to the injured cells without FLP treatment and 50 ​μg/mL FLP-treated cells (Fig. 2a). The mean fluro-intensity quantification was significantly different between 50 ​μg/mL FLP-treated and 200 ​μg/mL FLP-treated cells in 72 ​h (0.63 ​± ​0.22 versus 0.11 ​± ​0.06, P ​< ​0.001) (Fig. 2g), while 200 ​μg/mL FLP-treated fluro-intensity showed no differences with 50 ​μg/mL FLP-treated group in 24 and 48 ​h (Fig. 2e and f). The results confirm that 200 ​μg/mL of FLP ameliorated the survival of the IL-1β-induced chondrocyte injury instead of 50 ​μg/mL FLP.

### 2.5 Detection of intracellular oxidative stress

**Fig. 3:**
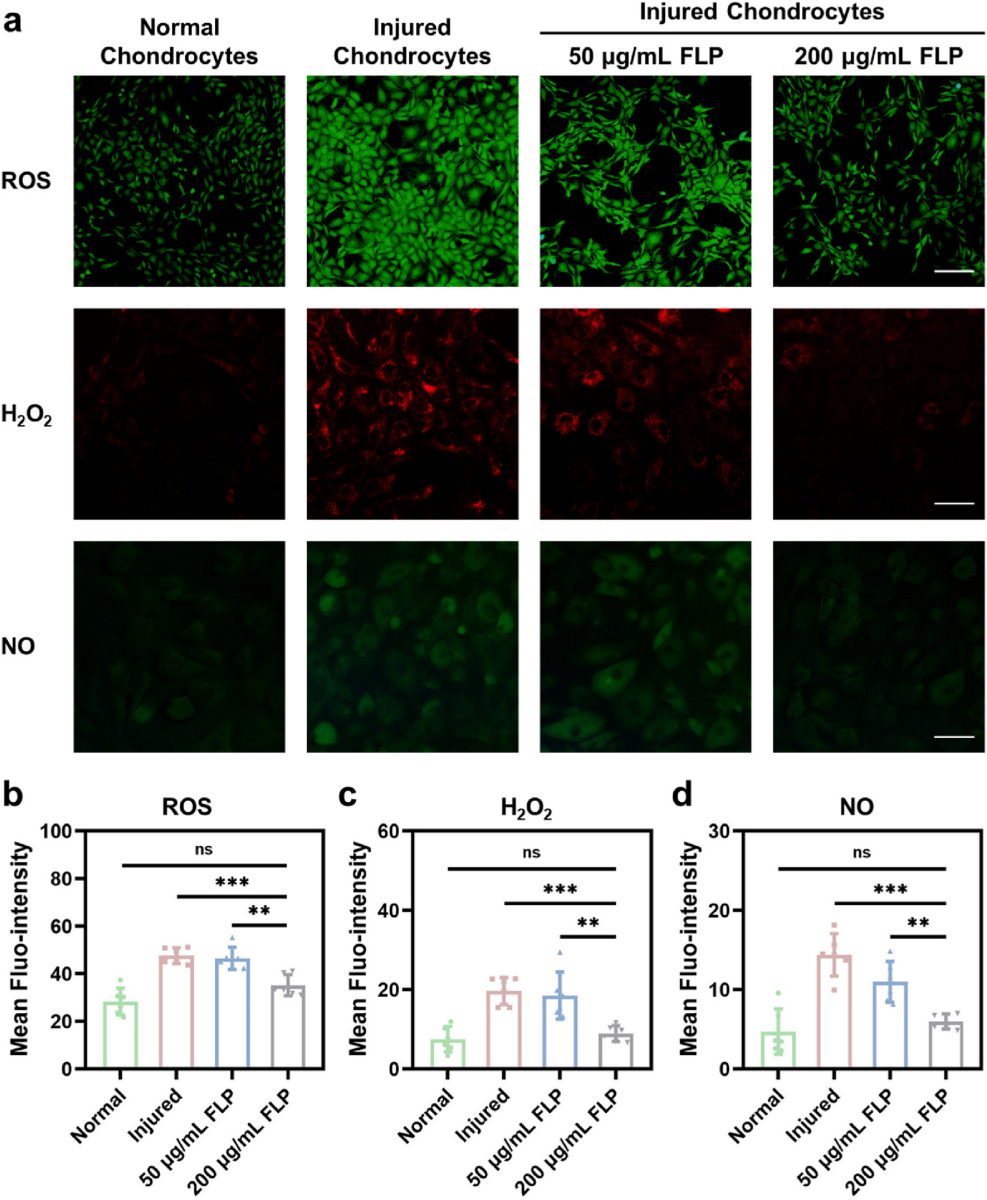
The detection of the intracellular contents of ROS, H_2_O_2_ and NO in the chondrocytes. a) ROS, H_2_O_2_ and NO that respectively stained with the specific probes (Scale bar for ROS, 200 ​μm; Scale bars for H_2_O_2_ and NO, 50 ​μm). b-d) Mean fluorescence intensity of ROS, H_2_O_2_ and NO (n ​= ​6). ∗∗P ​< ​0.01, ∗∗∗P ​< ​0.001 and ns meant no significance. All data were presented as mean ​± ​SD.

The free radical contents as ROS, H_2_O_2_ and NO after FLP co-culturing were further visualized by incubating with their specific probes. Compared with the normal chondrocytes, the ROS, H_2_O_2_ and NO markedly overexpressed in the injured chondrocytes. Importantly, high levels of these oxidative stress-associated substances not only result in cellular toxicity to the adjacent normal chondrocytes, but act as the pro-inflammatory factors that lead to various immune-related responses in the focal microenvironments [36]. Hence, it is of great significance to effectively prohibit free radical production. With treatment of 200 ​μg/mL FLP, the levels of ROS, H_2_O_2_ and NO were significantly attenuated in the injured chondrocytes, which were respectively mitigated more than that in 50 ​μg/mL FLP-treated cells (Fig. 3a). Specifically, the mean ROS fluro-intensity was 35.22 ​± ​4.45 for 200 ​μg/mL the FLP-treated chondrocytes, which significantly decreased by 26.04% of the injured chondrocytes (P ​< ​0.001) and 24.31% of 50 ​μg/mL the FLP-treated cells (P ​< ​0.01), respectively (Fig. 3b). Meanwhile, the mean fluro-intensity of the H_2_O_2_ addition for 200 ​μg/mL FLP-treated chondrocytes was 8.95 ​± ​2.00, reducing by 54.52% of the injured chondrocytes (P ​< ​0.001) and 51.65% of the 50 ​μg/mL FLP-treated cells (P ​< ​0.01), respectively (Fig. 3c). Similarly, the NO content also showed a marked suppression in the 200 ​μg/mL FLP-treated chondrocytes as 5.99 ​± ​0.96 in contrast to the other two groups of cells, of which the NO fluro-intensity were 14.40 ​± ​2.67 (P ​< ​0.001) and 11.00 ​± ​2.59 (P ​< ​0.01), respectively (Fig. 3d). Additionally, the inhibition rates of O_2_^•−^ in the supernatant from the cracked injured chondrocytes elevated along with the increased concentration of FLP from 10 ​μg/mL to 200 ​μg/mL (Fig. S2), which further proved the O_2_^•−^ scavenging efficacy of FLP *in vitro*. Besides, the *in vitro* total antioxidant capacity reached equivalently 1.14 ​mM Trolox when the FLP concentration was 200 ​μg/mL (Fig. S3), indicating that FLP was capable of clearing the excessive oxidative substances. Conclusively, 200 ​μg/mL of FLP massively depleted the production of ROS, H_2_O_2_, NO and O_2_^•−^, thus enabling the survival of the injured chondrocytes.

### 2.6 Analysis of inflammatory indicators by PCR

As shown in Fig. S4, the gene expressions of the inflammatory indicators including TNF-α, IL-1α and IL-1β significantly elevated in the injured chondrocytes than the normal cells. After treated with 200 ​μg/mL FLP, compared with the untreated injured cells, the expression fold changes of TNF-α, IL-1α and IL-1β returned to (14.88 ​± ​0.46), (2128.38 ​± ​134.36) and (9215.10 ​± ​776.59) from (22.83 ​± ​3.15), (3364.74 ​± ​414.65) and (11976.86 ​± ​522.02), respectively (all P ​< ​0.01). The restraining of the inflammatory indicators in the cells treated by 50 ​μg/mL FLP was also inferior to 200 ​μg/mL FLP. These above results implied that the inflammatory response was relieved with the treatment of 200 ​μg/mL FLP.

### 2.7 Cartilage protection efficacy *in vivo*

**Fig. 4:**
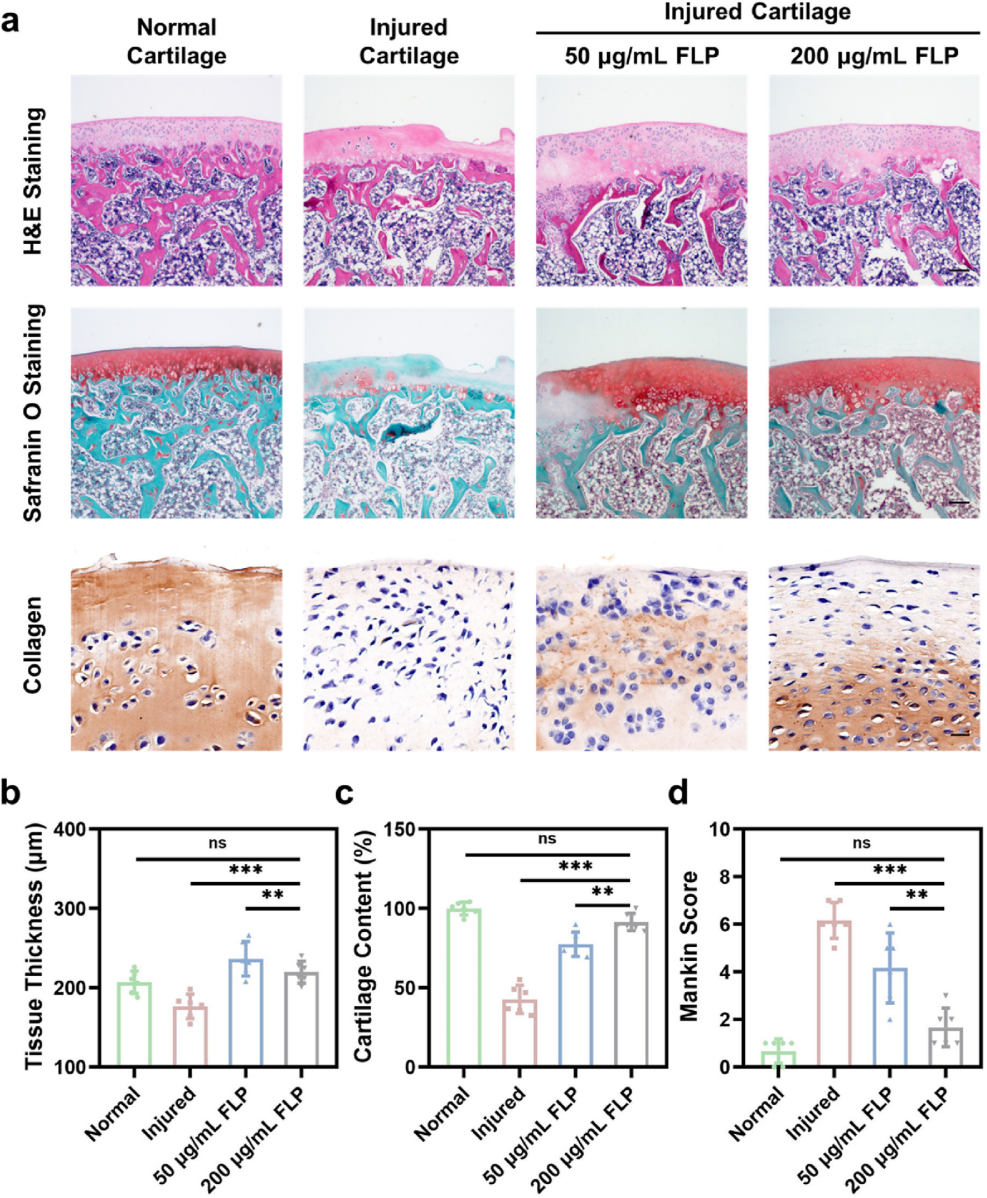
*In vivo* evaluation of the cartilage protection capability of FLP. a) H&E and Safranin O staining for the cartilage tissue sections (Scale bars, 200 ​μm) and immunohistochemical staining for type II collagen of the representative cartilage tissue (Scale bar, 20 ​μm). b) Cartilage tissue thickness measurement (n ​= ​6). c) Cartilage content estimation based on the Safranin O staining (n ​= ​6). d) Mankin histological scoring for the structure and quality of the cartilage (n ​= ​6). ∗∗P ​< ​0.01, ∗∗∗P ​< ​0.001 and ns meant no significance. All data were presented as mean ​± ​SD.

In this study, a preciously-verified rat model of osteoarthritis was established with the intra-articularly injected sodium iodoacetate [[37], [38], [39]], of which the cartilage tissue was sectioned and stained. As depicted in Fig. 4a, the osteoarthritic injured cartilage induced by IL-1β notably degenerated, in which a distinguishable gap was marked by H&E staining. Besides, the Safranin O-stained section showed the reduced red pigmentation, suggesting the reduction of glycosaminoglycan. Compared with 50 ​μg/mL FLP-treated cartilage, there was a smoother cartilage surface and a more orderly layer of continuously-stained glycosaminoglycan that were close to the structure of the normal cartilage with the treatment of 200 ​μg/mL FLP. More precisely showed in Fig. 4b, the tissue thickness was measured from the surface to the tidemark of cartilage and the average intensity of the cartilage content was calculated. The thickness of 200 ​μg/mL FLP-treated cartilage was 219.80 ​± ​13.88 ​μm versus 207.00 ​± ​14.20 ​μm of the normal cartilage (P ​> ​0.05), which implied that the injured cartilage thickness restored to normal size upon FLP treatment. Furthermore, the average cartilage content was 91.48% ​± ​5.48% of 200 ​μg/mL FLP, as opposed to 42.76% ​± ​8.92% (P ​< ​0.001) of the injured cartilage and 77.51% ​± ​7.72% (P ​< ​0.01) of 50 ​μg/mL FLP-treated cartilage (Fig. 4c). Moreover, for the histological analysis of Mankin score in Fig. 4d and 200 μg/mL FLP-treated cartilage exhibited a total score as 1.67 ​± ​0.82, showing significant differences to the injured cartilage (P ​< ​0.001) and 50 ​μg/mL FLP-treated cartilage (P ​< ​0.01). Above results verify that the superb protecting capability of 200 ​μg/mL FLP for the osteoarthritic injured cartilage, which was characterized by the preservation of the intact continuous layer of glycosaminoglycan.

Inspired by the encouraging results of glycosaminoglycan restoration *in vivo*, the efficacy of FLP for cartilage collagen protection was further measured, and immunohistochemical staining for type II collagen was also conducted. As displayed in Fig. 4a, the type II collagen staining was uniform distributed in the normal cartilage tissue, while the injured cartilage exhibited the rare staining. After the treatment of 50 ​μg/mL FLP, a small amount of type II collagen was disorderly preserved, of which the staining was apparently weakened than that of 200 ​μg/mL FLP-treated cartilage. More importantly, the even formation of collagen inside cartilage further revealed 200 ​μg/mL of FLP was superior for the protection of the injured cartilage. Besides, the biosafety of FLP for *in vivo* study was also evaluated by the H&E-staining of major organs including liver, kidney, spleen, intestine, heart, and lung, which demonstrated no significant pathological lesions in these groups (Fig. S5). Therefore, 200 ​μg/mL of FLP with approved biosafety would ameliorate the osteoarthritic injured cartilage by preserving the glycosaminoglycan and type II collagen, which were the main components of articular cartilage extracellular matrix.

## 3 Conclusions

In summary, the present study reports a novel therapeutic treatment for cartilage protection in osteoarthritis by intra-articular FLP delivery. 200 ​μg/mL FLP has been demonstrated to well-restore the cell viability of the injured chondrocytes to that of normal cells. Notably, this effect is achieved by diminishing oxidate-associated molecules such as ROS, H_2_O_2_ and NO. Furthermore, 200 ​μg/mL FLP can protect both glycosaminoglycan and type II collagen in the osteoarthritic cartilage tissue against obvious diminishment *in vivo*. Overall, the fabricated FLP possesses great potential for articular cartilage protection via mitigating the loss of extracellular matrix including glycosaminoglycan and type II collagen, which presents a promising opportunity for the articular cartilage repair and regeneration.

## 4 Material and methods

### 4.1 Synthesis and characterization of FLP

FLP was synthesized by the ultrasonication-assisted mechanical exfoliation method. In brief, 100 ​mg bulk black phosphorus crystal was dispersed in 100 ​mL 1-Methyl-2-pyrrolidinone (Sigma). The mixture was then put in an ultrasonicator (Scienta) at 600 ​W for 18 ​h, while using an ice-water bath. The ultrasound probe worked 3 ​s with an interval of 2 ​s. After ultrasonication, the solution was centrifuged at 1, 000 ​rpm for 5 ​min to remove any non-exfoliated bulk black phosphorus. Then, the supernatant was centrifuged at 13, 000 ​rpm for 10 ​min to get the precipitate and washed with ethanol for 3 times.

The characterization of FLP were achieved with transmission electron microscopy (TEM), high-resolution scanning transmission electron microscopy (HR-STEM) and energy dispersive X-ray spectroscopy (EDS), which were provided by the JEM-2100F field emission electron microscope at 200 ​kV (JEOL). Atomic force microscope (AFM) was provided by the Dimension ICON (Bruker). The quantitative analysis of sample elements was measured by an inductively coupled plasma-optical emission spectrometry (ICP-OES, Agilent). UV–vis–NIR absorbance spectra were collected by a UV–vis–NIR spectrometer (Shimadzu). The electron spin resonance (ESR) spectra were obtained with the Bruker E500 electron paramagnetic resonance spectrometer.

### 4.2 Electron spin resonance (ESR) measurement

Hydroxyl radicals were produced through the Fenton reaction. 5,5-dimethyl-1-pyrroline N-oxide (DMPO, Adamas) was used as the spin trapping agent. To study the ^•^OH-scavenging capacity of the product, FeSO_4_, DMPO, 30% H_2_O_2_ and FLP (100 ​μg/mL) were mixed in sequence to monitor the change of the relative peak intensity in the ESR spectra of the DMPO-OH^•^.

### 4.3 O_2_^−^ scavenging ability of FLP

The O_2_^•-^ scavenging effectiveness of FLP were evaluated by the commercial SOD assay kit. In brief, FLP with the concentrations of 10 μg/mL, 20 μg/mL, 40 μg/mL, 80 μg/mL, 100 μg/mL, 150 μg/mL and 200 ​μg/mL were added into the prepared detection reagent. After about 30 ​min incubation, the absorbance of samples at 450 ​nm were detected by microplate spectrophotometer.

### 4.4 ABTS radical scavenging assay

The antioxidant capacity of FLP was tested based on the reduction of ^•^ABTS ​+ ​radicals through the total antioxidant capacity assay kit (Beyotime). Briefly, ABTS was oxidized to generate ABTS radical cation (^•^ABTS+). Then, FLP with the concentrations of 10 ​μg/mL, 20 ​μg/mL, 40 ​μg/mL, 60 ​μg/mL, 80 ​μg/mL and 100 ​μg/mL was added into the detection reagent, while monitoring the absorbance at 734 ​nm after 15 ​min incubation. Trolox was set as the standard for evaluating the antioxidant levels of FLP.

### 4.5 H_2_O_2_ scavenging ability of FLP

H_2_O_2_ consumption capability of FLP was assessed by monitoring the change of characteristic absorption of H_2_O_2_ at 240 ​nm. Briefly, 100 ​μg/mL FLP and 20 ​mM ​H_2_O_2_ were mixed and reacted for period of time, while monitoring the change of absorption at 240 ​nm at varied time points.

### 4.6 Isolation of articular chondrocytes

Animals in this study were provided by Department of Laboratory Animal Science of Shanghai Tenth People's Hospital, Tongji University School of Medicine, and all experiments involving the animals were authorized by Animal Care and Use Committee. Chondrocytes were isolated and prepared by degrading the articular cartilage of a mature Sprague-Dawley (SD) rat, in which the cartilage tissue was digested with type II collagenase (Sigma) for 4 ​h at 37 ​°C. The second generation of the chondrocytes were used in the cellular experiments.

### 4.7 Induction and identification of the injured chondrocytes

The isolated articular chondrocytes were stimulated with the rat interleukin-1β (IL-1β, Sino Biological) for at least 24 ​h to prepare the osteoarthritic injured chondrocytes. Then, immunofluorescent staining was applied for the identification of CD54 molecule with its specific antibody (Santa Cruz). The semiquantitative expression of CD54 was calculated and compared between the normal chondrocytes and the injured cells.

### 4.8 CCK-8 assay for determining FLP concentration

Different concentrations of FLP were diluted from 200 ​μg/mL to 0.01 ​μg/mL, and they were co-cultured with the injured chondrocytes in a 96-well cell culture plate (1 ​× ​10^4^ ​cells per well) for 48 ​h. CCK-8 reagent (Dojindo) was mixed with the culture medium at a volume ratio of 1: 9, following with incubation for 2 ​h at 37 ​°C. The absorbance was measured at 450 ​nm using a multi-mode reader (BioTek), and the suitable concentrations of FLP (50 ​μg/mL and 200 ​μg/mL) were acquired for the subsequent tests.

### 4.9 Chondrocyte survival analysis with live/dead staining

The normal and injured chondrocytes were seeded in a 24-well culture plate at a density of 1 ​× ​10^5^ ​cells per well and cultured overnight. 50 ​μg/mL and 200 ​μg/mL FLP were then co-cultured with these chondrocytes for 48 ​h at 37 ​°C, respectively. The cells were washed and fixed with paraformaldehyde, followed by the incubation with fluorescent live/dead staining (Calcein/PI assay kit, Beyotime). The region of interest was captured by a fluorescent microscope (Olympus), and the mean intensity of the fluorescence (mean fluro-intensity) was measured with Image J software.

### 4.10 Intracellular changes of oxidative stress-associated compounds

Chondrocytes were pre-seeded in a 24-well culture plate (1 ​× ​10^5^ ​cells per well) and cultured with 50 ​μg/mL and 200 ​μg/mL FLP for 48 ​h. Then, intracellular ROS, H_2_O_2_ and NO in the injured chondrocytes were respectively detected with ROS assay kit (Beyotime), H_2_O_2_ probe (MKBio) and NO probe (MKBio), which were further visualized by the fluorescent microscope and captured for the region of interest to calculate the mean fluro-intensity.

### 4.11 *In vitro* SOD test

Cells were seeded in a 6-well culture plate with a density of 1 ​× ​10^5^ ​cells per well and cultured until the cell confluence was 80%. The cell culture medium was replaced with the different concentrations of FLP, and then cultured for 48 ​h at 37 ​°C. Then, the treated cells were collected for cracking with a homogenizer and centrifuged at 10, 000 ​rpm for 15 ​min to preserve the supernatant. WST-1 working solution (1 ​mL) in the assay kit was diluted with 19 ​mL buffer solution, and 15 ​μL enzyme solution was mixed with 2.5 ​mL dilution buffer. 20 ​μL sample and 20 ​μL diluted enzyme solution were mixed with 200 ​μL diluted WST-1 working solution. After incubated for 20 ​min at 37 ​°C, the absorbance at 450 ​nm was measured by a multi-mode reader (BioTek).

### 4.12 *In vitro* ABST test

The cells were prepared following the similar method in the previous SOD test. After treated with different concentrations of FLP, the supernatant was collected from the cracked cells. The ABTS working solution (200 ​μL) was mixed with 10 ​μL sample solution or Trolox standard solution to measure the A734 after incubation at room temperature for 5 ​min.

### 4.13 PCR analysis

The cells were treated with FLP, and then analyzed by quantitative Polymerase Chain Reaction (PCR) for the cytokine contents of Tumor Necrosis Factor-α (TNF-α), Interleukin-1α (IL-1α) and Interleukin-1β (IL-1β). The primer information used in the PCR test was provided in the following Table 1.

**Table 1:** PCR primer gene sequences.

Gene	Forward primer	Reverse primer
GAPDH	CCTCGTCCCGTAGACAAAATG	TGAGGTCAATGAAGGGGTCGT
TNF-α	GTGCCTATGTCTCAGCCTCTTCTC	GTTTGTGAGTGTGAGGGTCTGG
IL-1α	CGGGTGACAGTATCAGCAACGT	TGACAAACTTCTGCCTGACGAG
IL-1β	AAATGCCACCTTTTGACAGTGA	AAAGAAGGTGCTCATGTCCTCATCC

### 4.14 The animal model of osteoarthritic injured cartilage

24 rats in total were selected to establish the animal model of osteoarthritis, and were randomly assigned into 4 experimental groups (6 rats per group). With the general anesthesia using intra-peritoneal pentobarbital sodium (5 mg/100 ​g), the skin surface of intra-articular injection site was carefully disinfected with medical iodophor. Then, 1 ​mg sodium iodoacetate (Sigma) in 50 ​μL volume of physiological saline was injected into the articular cavity to produce osteoarthritic changes of the articular cartilage. For the therapeutic study of the injured cartilage, 50 ​μg/mL and 200 ​μg/mL FLP were additionally injected at a volume of 50 ​μL.

### 4.15 *In vivo* cartilage protective efficacy evaluation

After FLP treatment for 6 weeks, the rats were sacrificed. The distal femurs were fixed with paraformaldehyde, decalcified with EDTA decalcification kit (Ribology) and sectioned into 5-μm slides. Then, hematoxylin and eosin (H&E), Safranin O and type II collagen immunohistochemical staining were used for the verification of the cartilage tissue structure and quality. The cartilage tissue thickness from the surface to the tidemark of the retained cartilage and the cartilage content of the injured cartilage referring to the normal tissue were separately measured. Besides, Mankin histological scoring system was utilized for the semiquantitative evaluation of the cartilage.

### 4.16 Statistical analysis

In this work, the experiments were duplicated for at least 3 times. All statistical data were showed as mean ​± ​standard deviation (SD), and the significant differences were analyzed with GraghPad Prism software. Once the P value was less than 0.05, the difference between two groups was considered to be statistically significant (∗P ​< ​0.05, ∗∗P ​< ​0.01 and ∗∗∗P ​< ​0.001). Ns meant that there were no significances.

## Authors’ contributions

**XY Zhang**, **YL You** and **H Lin** design this research. **XY Zhang**, **YL You** and **YY Sun** conducted the experiments. **XY Zhang** and **YL You** analyzed the results. **XY Zhang** and **YL You** wrote the manuscript. **H Lin**, **M Zong** and **JL Shi** reviewed the manuscript. All authors read and approved the final manuscript.

## Availability of data and materials

All data generated or analyzed during this study are included in this published article.

## Ethics approval and consent to participate

All animal studies were confirmed to the guidelines by the Animal Care Ethics Commission of Shanghai Tenth People's Hospital, Tongji University School of Medicine (SHDSYY-2020-Z0026).

## Declaration of competing interest

The authors declare that they have no known competing financial interests or personal relationships that could have appeared to influence the work reported in this paper.

## Data Availability

Data will be made available on request.
