# Supplementary material for: Catalytic anti-oxidative stress for osteoarthritis treatment by few-layered phosphorene
Source: Mater Today Bio. 2022 Oct 15;17:100462. doi: 10.1016/j.mtbio.2022.100462 (PMC9619373; doi:10.1016/j.mtbio.2022.100462)
Supplement: Multimedia component 1 [file mmc1.docx]

***Supplementary Information***

**Catalytic Anti-Oxidative Stress for Osteoarthritis Treatment by Few-Layered Phosphorene**

*Xingyu Zhang^1†^, Yanling You^3†^, Yaying Sun^4†^, Xiang Guo^5^*, Han Lin^3^*, Ming Zong^2^*, Jianlin Shi^3^*

1. Department of Sports Medicine, Shanghai General Hospital, Shanghai Jiao Tong University School of Medicine, Shanghai 200080, P. R. China.

2. Department of Clinical Laboratory, Shanghai East Hospital, Tongji University School of Medicine, Shanghai, 200120, P. R. China.

3. State Key Laboratory of High Performance Ceramics and Superfine Microstructure, Shanghai Institute of Ceramics Chinese Academy of Sciences, Shanghai 200050, P. R. China.

4. Department of Sports Medicine, Huashan Hospital, Fudan University, Shanghai 200040, P. R. China.

5. Department of Orthopedics, Second Affiliated Hospital of Naval Medical University, Shanghai, 200003, P. R. China.

†Xingyu Zhang, Yanling You and Yaying Sun contributed equally to this manuscript.

*Corresponding authors: gx9601074@smmu.edu.cn; linhan@mail.sic.ac.cn; zongming@tongji.edu.cn

**Additional figures**


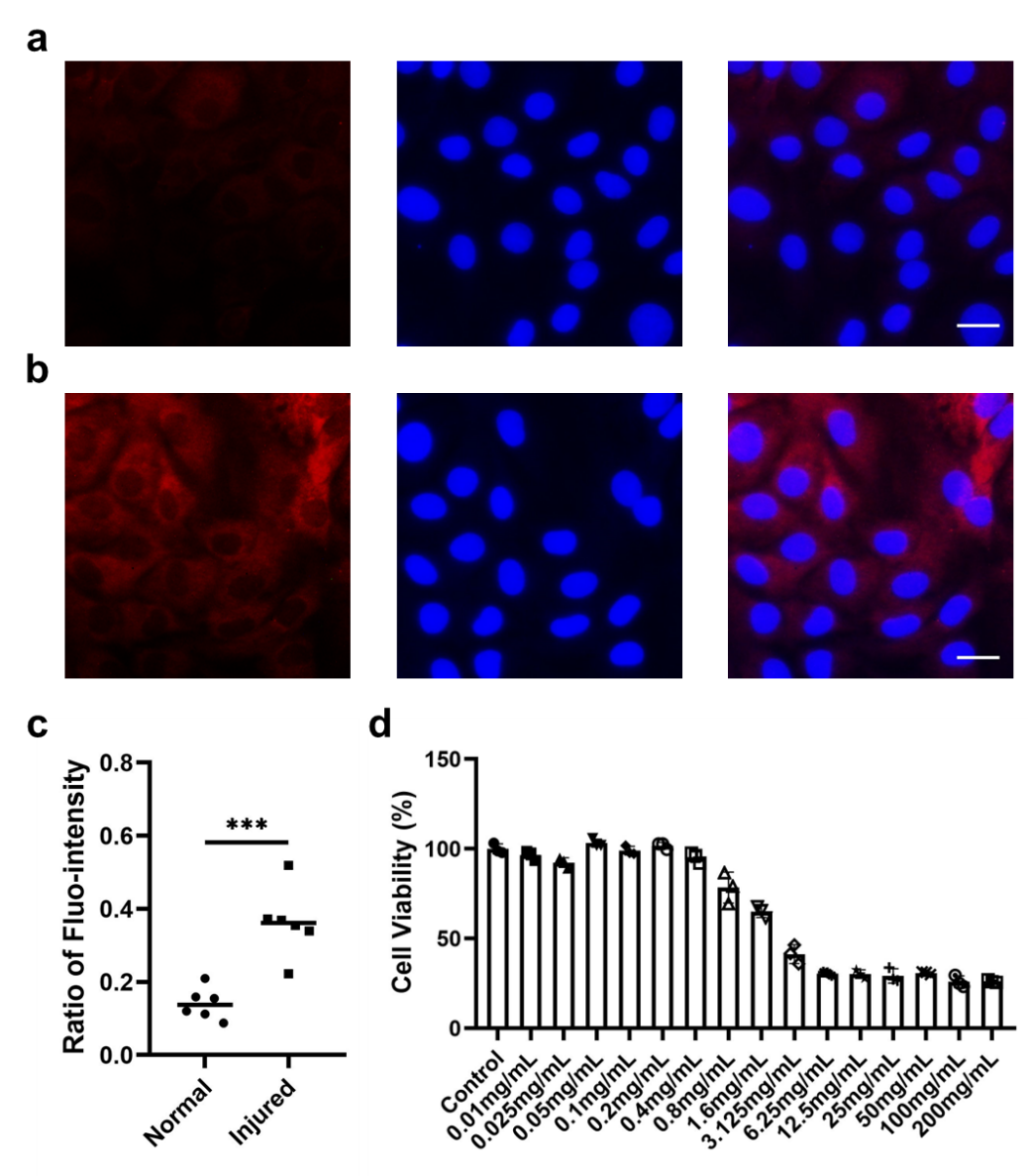


**Figure S1.** Chondrocyte activation and CCK8 assay for FLP concentration screening (Scale bars, 20 μm). a) The expression of CD54 in the normal chondrocytes. b) CD54 expression in the injured chondrocytes after IL-1β stimulation. c) Ratio of the CD54/DAPI fluorescence intensity (n = 6, ***P < 0.001). d) Cell viabilities of the injured chondrocytes with the treatment of different FLP concentrations (n = 3). All data were presented as mean ± SD.


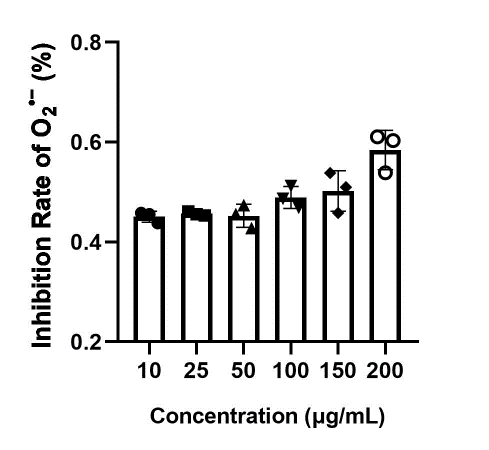


**Figure S2.** The evaluation for the O_2_^•−^ scavenging capability after FLP treatment (n = 3). All data were presented as mean ± SD.


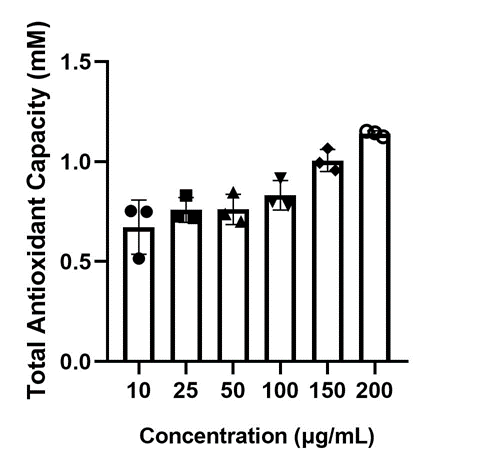


**Figure S3.** The evaluation for the equivalent Trolox (Total antioxidant capacity) of different concentrations of FLP (n = 3). All data were presented as mean ± SD.

**Figure S4.** The PCR analysis of the inflammatory indicators including TNF-α, IL-1α and IL-1β (n = 3, *P < 0.05, **P < 0.01 and ***P < 0.001). All data were presented as mean ± SD.


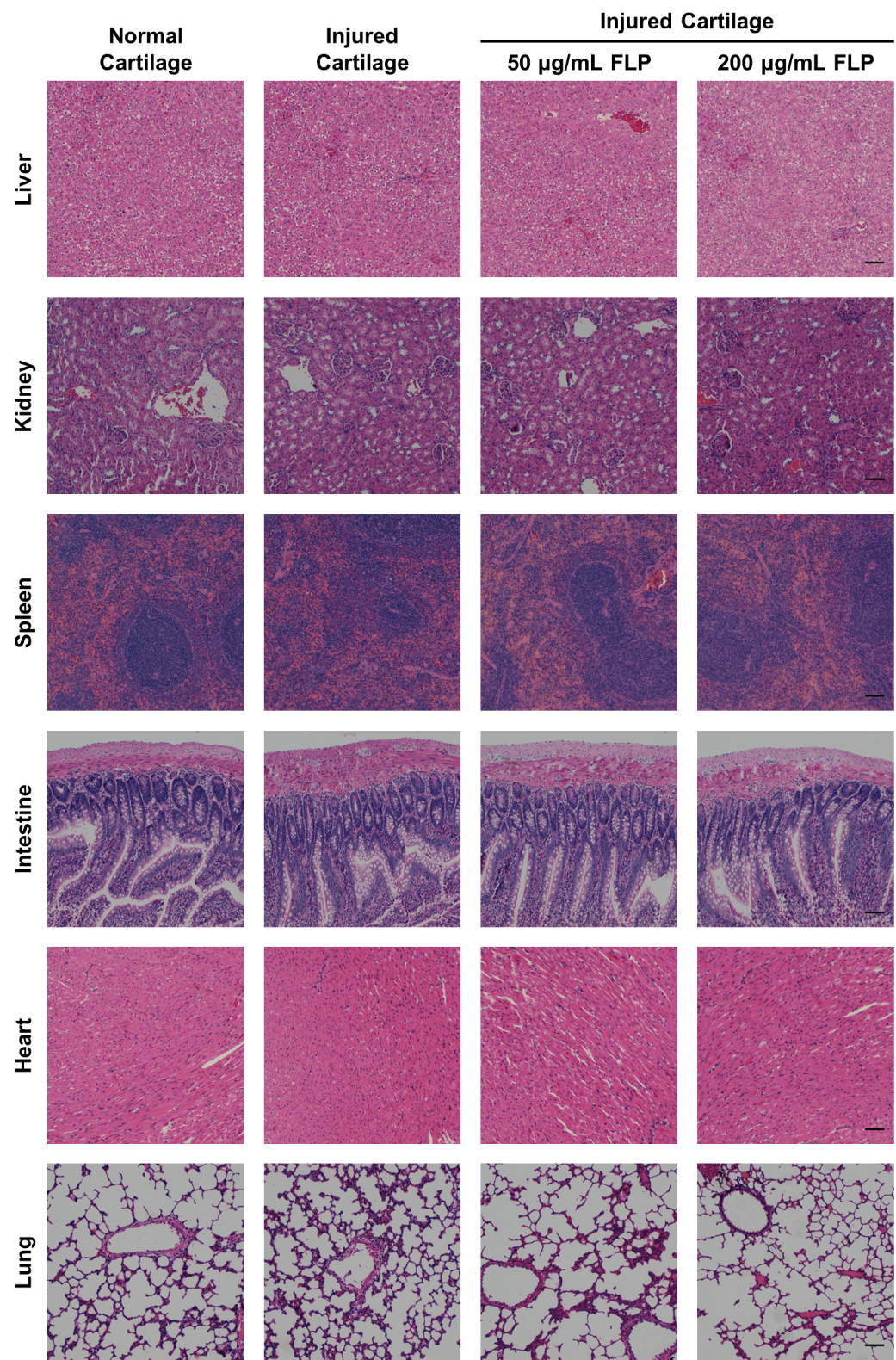


**Figure S5.** The *in vivo* biosafety property evaluation of FLP by the H&E-stained sections of different organs (Scale bars, 200 μm).
